# Retirement and grandchild care in China: mental health consequences and policy implications

**DOI:** 10.1186/s12889-025-24774-x

**Published:** 2025-10-08

**Authors:** Yang Mou, Zijun Tan, Hongxian Nie

**Affiliations:** 1 School of Education Yunnan College of Business Management Kunming, Kunming, Yunnan Province 650106 China; 2 School of Early Childhood Education, Kunming Preschool Education College, Kunming, Yunnan Province 651700 China

**Keywords:** Retirement policy, Grandparental caregiving,Mental health, Intergenerational support, China, Older adults

## Abstract

**Background:**

In China’s rapidly aging society, retirement and grandchild caregiving are two critical life transitions for older adults. This study examines how these dynamics jointly relate to depressive symptoms among older adults and explores their implications for coordinating elder-care and childcare policies.

**Methods:**

Using data from the 2018 wave of the China Health and Retirement Longitudinal Study (CHARLS; ages 40–75; *N* = 7,730), we estimate OLS and IV–2SLS models. Gender-specific statutory retirement ages (men 60, women 55) serve as instruments for retirement. Depressive symptoms are measured using the standardized CES-D10 scale. Covariates include parent–child satisfaction, caregiving status and intensity, health status, marital status, household size, education, urban/rural residence, and age. Robustness checks include weak-IV-robust inference, a restricted 55–65 age bandwidth, and a supplementary male-only RDD/IV analysis.

**Results:**

OLS results suggest retirees report fewer depressive symptoms, whereas IV estimates indicate that individuals “nudged” into retirement at statutory ages experience higher depression. Instrument strength is borderline in the full sample, stronger within the 55–65 age range, but weak in the male-only RDD/IV. Grandchild caregiving shows a dual pattern: providing any care is associated with lower depression, but supplementary spline analyses reveal that depressive symptoms rise significantly once weekly caregiving exceeds 40 h. Interaction analyses indicate that higher education and better self-rated health mitigate the adverse association between retirement and depression. Parent–child satisfaction consistently predicts better mental health across models, underscoring its protective role.

**Conclusion:**

Naïve associations may reflect favorable selection and post-retirement lifestyle adjustments, whereas IV estimates highlight the psychological costs of abrupt, rule-based retirement. Large IV estimates should be interpreted cautiously as local rather than precise effects. Policy reforms delaying retirement should be accompanied by phased exit options, routine psychological screening and counseling, and expanded investment in affordable childcare and respite services to alleviate dual pressures on older adults and foster intergenerational harmony.

**Supplementary Information:**

The online version contains supplementary material available at 10.1186/s12889-025-24774-x.

## Introduction

China is undergoing a profound demographic shift. By the middle of this century, it is expected that nearly one-third of its citizens will be aged 60 or above. To address the growing economic strain posed by an aging labor force, policymakers have implemented delayed retirement strategies. While these measures aim to sustain fiscal balance, they may unintentionally increase mental burdens on older adults, particularly those who are also serving as caregivers for their grandchildren.

Concurrently, family life in China is being reshaped by rapid urbanization and internal migration. These social shifts have resulted in more grandparents stepping in as the primary providers of childcare. Although this role can offer emotional rewards and strengthen family ties, it also brings with it significant physical and psychological challenges for older adults.

Past research has primarily treated retirement and caregiving as separate phenomena, often producing conflicting findings regarding the relationship between them and mental health. However, few studies have examined how these two experiences interact, particularly in the context of China’s evolving economic policies and cultural norms. What remains underexplored is how stepping away from employment and assuming intensive caregiving duties intersect—particularly against China’s specific economic pressures and social policies.

Importantly, while our study conceptualizes retirement as a discrete transition based on available survey data, we acknowledge the growing recognition in the literature that later-life employment is not always linear or permanent. Older adults may re-enter the labor force after retirement or retire gradually, and the psychological implications of exiting versus re-entering employment may differ substantially. This dynamic view of retirement has been highlighted in recent work by Park & Kim (2025) and Park, Park, & Kim (2024), which emphasize the complexity and heterogeneity of retirement pathways. Our approach focuses on self-reported retirement status at a specific point in time, which, while limited in capturing dynamic transitions, still yields important insights into the mental health implications of formal labor market withdrawal in the Chinese context [1, 2].

Moreover, while intergenerational support holds cultural significance, its association with the intersection of retirement, caregiving, and mental health remains insufficiently explored.This study seeks to fill these gaps by addressing the following research questions:

1. How is retirement status associated with the mental health of older adults in China?
2. What is the relationship between caregiving intensity for grandchildren and psychological well-being?
3. Can intergenerational support buffer the mental health burdens associated with retirement and caregiving?

Utilizing data from the China Health and Retirement Longitudinal Study (CHARLS) and an instrumental variable approach, this study provides empirical insights into the complex relationships between retirement, caregiving, and intergenerational support. Our findings offer valuable guidance for policymakers aiming to balance labor market objectives with the caregiving needs of families and the mental health of older populations.

### Retirement and mental health: contradictory findings

The relationship between retirement and mental health has been widely debated, yielding mixed results. with some studies suggesting that retirement can enhance psychological well-being, particularly in the initial years, as retirees often experience relief from work-related stress, leading to improvements in self-esteem, life satisfaction, and overall mental health [3, 4]. These benefits are most evident among those who retire voluntarily and view the transition positively [5].

While the positive associations of retirement may initially seem evident, they tend to diminish over time.Many individuals who remain retired for extended periods often grapple with emotional difficulties such as loneliness, a diminished sense of purpose, and social withdrawal—each of which can take a toll on mental well-being [6, 7]. Notably, the mental health outcomes of retirement are closely linked to how that retirement comes about. People who exit the workforce involuntarily—for reasons like sudden job loss or chronic illness—tend to report higher levels of psychological distress, including heightened anxiety and depressive symptoms [8, 9]. In contrast, those who make the decision to retire on their own terms often fare better emotionally [10, 11].

Financial conditions add another layer of complexity. A lack of adequate income during retirement can intensify emotional struggles, particularly for those with minimal savings or unstable pensions [3, 4]. However, older adults who remain socially active and maintain strong interpersonal connections are often more resilient, underscoring the buffering related to social engagement and community participation [6, 12].

Cultural expectations also shape the retirement experience. In contexts where professional life is deeply entwined with self-identity, stepping away from work may trigger a deep sense of disorientation or emotional void [5, 13]. Taken together, while retirement can offer relief and psychological benefits in the short term, its longer-term impacts are shaped by a combination of voluntariness, economic stability, and opportunities for meaningful social connection [1, 2, 11].

### Grandchild caregiving and mental health: the association of intensity

The extent to which grandparents are involved in childcare significantly shapes their emotional well-being. When caregiving becomes too demanding, many older adults report heightened stress, emotional fatigue, and even physical symptoms like disrupted sleep or recurring headaches [14]. In contrast, those who provide care in more moderate or occasional roles often maintain better psychological balance. These findings suggest that there may be a critical point at which caregiving shifts from fulfilling to burdensome [15].

Nonetheless, grandchild caregiving is not inherently detrimental. For many seniors, participating in their grandchildren’s lives offers emotional satisfaction, a renewed sense of purpose, and a meaningful role within the family unit. Feeling appreciated can help reduce feelings of loneliness or sadness and bolster psychological resilience [16]. However, when this role dominates daily life, the demands can become overwhelming. Caregivers in these situations frequently report emotional strain, including chronic anxiety and persistent fatigue—often intensified by ongoing concerns about their grandchildren’s future and a lack of personal time or space [17, 18].

In such circumstances, access to strong social connections and effective personal coping strategies becomes especially important. These protective factors can act as emotional buffers, helping to mitigate the psychological costs of intense caregiving commitments.Grandparents who maintain strong relationships and receive emotional support from family and friends are more likely to report better mental health [19, 20]. Additionally, financial and reciprocal support from adult children can ease the psychological burden. When caregiving is coupled with both emotional and financial support from other family members, it helps reduce depressive symptoms and shifts caregiving from a solitary responsibility to a shared family duty [21].

While intergenerational support generally benefits caregivers, imbalances in these exchanges can lead to burnout. Open and honest communication between family members about caregiving roles, expectations, and boundaries is essential to ensure that caregiving remains a rewarding experience without leading to exhaustion [22].

### Delayed retirement and its psychological impact

Delayed retirement has become an increasingly important topic of research, yet its mental health related to are multifaceted and often contradictory. For some individuals, staying in the workforce provides social connections and a continued sense of purpose. However, for others, working beyond the typical retirement age can lead to elevated psychological strain and a diminished sense of social status, especially when one’s identity is strongly tied to their professional role [23].

The mental health outcomes associated with retirement are closely shaped by when and how individuals retire. Those who have time to anticipate and prepare for the transition generally navigate it more smoothly, often reporting greater emotional stability than peers forced into retirement due to layoffs or challenging work conditions [24].

Another important factor is cognitive health. Remaining in intellectually stimulating roles may help preserve mental agility, but continued exposure to high-pressure environments can wear down cognitive reserves, potentially leading to mental fatigue later on [11].

For some, postponing retirement brings financial relief. Yet for others—especially those uncertain about their long-term income—delayed exit from the workforce can worsen anxiety and emotional distress [25]. With more people now choosing to extend their careers, there is an urgent need to reconsider retirement frameworks that accommodate diverse needs and avoid a one-size-fits-all approach [22].

Ultimately, while staying in the workforce may offer certain financial or social benefits, it can also come at a psychological cost. To support older adults through this complex transition, flexible retirement options that consider personal and economic realities are increasingly vital.

### The role of intergenerational support in mitigating psychological burdens

Support from younger generations can significantly lighten the emotional burden many older adults carry. Spending time with children or grandchildren—whether through daily activities, shared meals, or simply conversation—helps maintain a sense of connection that protects against social isolation, a key predictor of depression and anxiety in later life [26, 27].In addition to emotional support, practical assistance such as help with everyday tasks and financial contributions can greatly alleviate the challenges of aging, especially for those facing physical limitations [28].

Financial support from children is particularly important as older adults transition into retirement and face reduced income. This support alleviates economic stress and improves mental health outcomes [29]. In addition to practical and financial support, caregiving roles can provide a sense of purpose, enhancing self-worth and reducing depression, especially when grandparents feel needed by their grandchildren [14]. However, balance is crucial to prevent caregiver burnout.Effective communication between generations is key to ensuring caregiving remains fulfilling and does not lead to exhaustion associated with caregiving [20].

### Research gaps and future directions

Although considerable research has examined the individual associations of retirement and caregiving with mental health, few studies have explored how these two roles intersect, particularly in the Chinese context. Older adults often face the dual pressures of retirement and caregiving, yet the ways in which these experiences interact to shape psychological well-being remain insufficiently understood. Future research should therefore investigate the interplay between retirement and caregiving within China’s rapidly changing socio-economic environment.

In addition, the role of formal support services—such as respite care and community-based programs—deserves more attention, as such services may alleviate the burdens associated with intensive caregiving. Longitudinal data will also be essential for tracing the long-term mental health impacts of both retirement transitions and caregiving intensity.

As China’s demographic landscape continues to evolve, it is equally important to understand how intergenerational support systems adapt. Future studies should examine the diverse forms of intergenerational support and their implications for older adults’ mental health, with close consideration of cultural and policy contexts.

### Integrative conceptual framework

**Fig. 1:**
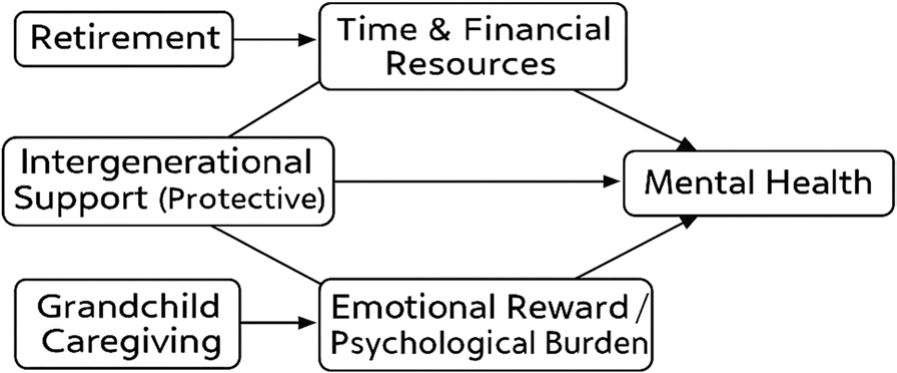
Integrative conceptual framework. Retirement reallocates time and financial resources, which may interact with grandchild caregiving to shape mental health outcomes. Intergenerational support functions as an independent protective factor, directly alleviating stress and fostering resilience, thereby mitigating the adverse psychological consequences of retirement or intensive caregiving

Previous research has typically examined retirement, grandchild caregiving, and intergenerational support in isolation, yet these domains are inherently interconnected. Retirement reallocates older adults’ time and financial resources, creating both opportunities and vulnerabilities. Grandchild caregiving constitutes a major competing demand: light involvement may enhance well-being through emotional rewards and a renewed sense of purpose, whereas intensive caregiving can impose psychological strain and reduce autonomy. Intergenerational support—both emotional and financial—functions as an independent protective factor that directly alleviates stress and fosters psychological resilience, thereby reducing depressive symptoms. We therefore conceptualize mental health in later life as the outcome of a dynamic interplay: (1) retirement alters the availability of time and economic resources, (2) caregiving intensity determines whether these resources become fulfilling or burdensome, and (3) intergenerational support provides an additional protective layer that promotes better mental health regardless of retirement or caregiving status (Fig. 1).

This framework aligns with stress-buffer theory, which emphasizes how supportive relationships mitigate the psychological costs of stress, and with role theory, which highlights how assuming or losing social roles such as worker, caregiver, or grandparent fundamentally shapes mental health.

## Method

We draw on the 2018 wave of the harmonised CHARLS individual-level data. Starting from roughly 20,255 respondents, we restrict the age range to 40–75 so that the sample spans the years just before and after the statutory retirement ages, leaving 7,825 observations. We then retain only those with complete information on retirement status, the standardized CES-D10 depression score, self-rated health status, satisfaction with parent–child relationships, whether grandchild care is provided and weekly caregiving hours, marital status, household size, years of schooling, urban or rural residence, and exact age (including a squared term to account for non-linear age effects). Applying listwise deletion yields a clean analytic (core) sample of 7,730 individuals—4,107 men and 3,623 women. Weekly grandchild-care hours are top-coded at 168; no imputation is performed, and any missing values lead to case-wise exclusion.

Table 1 reports the stepwise construction of the core cohort and the smaller “full” subsample used when pension receipt and intergenerational financial transfer variables (support to and from children) are required.

**Table 1:** Stepwise sample construction for the core analysis cohort

Step	Constraint (added cumulatively)	Sample size (N)
Initial sample	Respondents aged 40–75	7,825
Drop missing: Retirement Status	Non-missing (Retirement Status)	7,773
Drop missing: Standardized CES-D10 Depression Score	Non-missing (CES-D10 score)	7,733
Drop missing: Self-rated Health Status	Non-missing (Self-rated Health Status)	7,730
Drop missing: Parent–Child Relationship Satisfaction, Providing Grandchild Care, Caregiving Intensity, Marital Status, Household Size, Years of Schooling, Urban/Rural Residence, Age, Age Squared	All above non-missing (forms core analytic sample)	7,730
Drop missing: Pension Receipt	Non-missing (Pension)	6,947
Drop missing: Financial Support from Children (log)	Non-missing (log financial transfers from children)	5,562
Drop missing: Financial Support to Children (log)	Non-missing (log financial transfers to children)	2,657

Because including intergenerational financial transfers sharply reduces the analytic sample (down to about one-third of the core cohort), these variables are not incorporated into the main regression models. Given their substantive importance, however, we discuss them as a direction for future research using larger or pooled data sources.

### Weighting, complex-survey features, and inference

Descriptive statistics are unweighted. The main regression results in the text (Tables 4, 5, 6 and 7) are estimated without survey weights; p-values use heteroskedasticity-robust standard errors clustered at the primary sampling unit (PSU/community). This targets consistent slope estimates and accounts for within-PSU correlation.

As a robustness check, we also ran survey-weighted replications under a full complex-survey framework (weights, strata, PSU clustering, Taylor linearization); results are directionally unchanged (not shown; available on request).

In all IV models we use a single instrument (crossing the sex-specific statutory age threshold), so over-identification tests (e.g., Hansen’s J) are not applicable. We report the Kleibergen–Paap rk Wald F and supplement inference with Anderson–Rubin and Stock–Wright weak-IV-robust tests; first-stage diagnostics are summarized in Appendix Table A1.

### Variable definitions

#### Dependent variable

The primary outcome variable is depressive symptoms, measured using the 10-item Center for Epidemiologic Studies Depression Scale (CES-D10). This scale assesses depressive symptoms such as sadness, anxiety, and loss of interest among older adults. In CHARLS, the original CES-D10 scores range from 0 to 30, with higher values indicating more severe depressive symptoms. For comparability and ease of interpretation, we standardized the score (z-score), yielding a range of approximately –1.33 to 3.24 in our analytic sample.

#### Independent variables

- Retirement Status(Retire):A binary variable indicating whether the individual is retired (1 = retired, 0 = not retired). This variable captures the respondent's labor market exit status and serves as a key explanatory variable.
- Grandchild Caregiving (WPC):A binary variable representing whether the respondent provides caregiving for grandchildren (1 = provides care, 0 = does not provide care). This variable reflects the respondent's involvement in family caregiving roles.
- Caregiving Intensity (HTSPCH): A continuous variable indicating the number of hours per week the respondent spends caring for grandchildren. The variable ranges from 0 to 336 h, with extreme values above 168 h top-coded at 168 to maintain data plausibility and reduce outlier related to.Intergenerational Support.
- Parent–Child Relationship Satisfaction (Sati_Child): An ordinal variable measuring satisfaction with the parent–child relationship, ranging from 1 (very dissatisfied) to 5 (very satisfied), reflecting the intensity of emotional support.

#### Financial support

- Financial Support to Children (Log_Fcamt): A continuous variable representing the amount of financial support provided to children, measured as the log-transformed financial amount.
- Financial Support from Children (Log_Tcamt):A continuous variable representing the amount of financial support received from children, measured as the log-transformed financial amount.

#### Control variables

- Squared Age Term (c.age#c.age): The square of the individual’s age (c.age) is included as a continuous variable to account for non-linear age effects in the regression models.
- Self-Rated Health Status(SRH): An ordinal variable reflecting the individual’s subjective health perception, with a scale from 1 (very poor) to 5 (excellent).
- Pension Status (Pension):A binary variable indicating whether the individual receives a pension (1 = receives pension, 0 = does not receive pension).
- Marital Status (Marry):A binary variable indicating whether the individual is married (1 = married, 0 = not married).
- Family Size (Family_Size):A continuous variable indicating the number of household members, ranging from 1 to 13.
- Number of Grandchildren under 16 (grandchildu16): A continuous variable indicating the number of grandchildren under the age of 16, with a range from 0 to 14.

The definitions and measurement methods of all variables used in the analysis are summarized in Table 2.

**Table 2:** Variable definitions

Variable	Definition	Measurement
Standardized CES-D10 score	Standardized CES-D10 score, a measure of depressive symptoms in older adults. Assesses symptoms such as sadness, anxiety, and loss of interest	Continuous variable, range: −1.325 to 3.24. Higher scores indicate more severe depressive symptoms
Retirement Status	Indicator variable for whether the individual is retired	Binary variable (1 = retired, 0 = not retired)
Parent–Child Relationship Satisfaction	Satisfaction with the parent–child relationship	Ordinal variable (1 = very dissatisfied, 5 = very satisfied)
Grandchild Caregiving	Indicates whether the individual provides caregiving to grandchildren	Binary variable (1 = provides care, 0 = does not provide care)
Caregiving Intensity	Number of hours per week spent providing caregiving to grandchildren, reflecting caregiving intensity	Continuous variable, measured in hours per week, range 0–336; values above 168 top-coded at 168
Self-rated health status	Self-rated health status, reflecting the individual’s subjective health perception	Ordinal variable (1 = very poor, 5 = excellent)
Pension Status	Indicates whether the individual receives a pension	Binary variable (1 = receives pension, 0 = does not receive pension)
Financial Support to Children	Log-transformed amount of financial support provided to children	Continuous variable, log-transformed financial support amount
Financial Support from Children	Log-transformed amount of financial support received from children	Continuous variable, log-transformed financial support amount
The individual’s age	The individual’s age	Continuous variable, measured in years
Squared term of age to account for non-linear age effects	Squared term of age to account for non-linear age effects	Continuous variable, the square of c.age
Marital status	Marital status	Binary variable (1 = married, 0 = not married)
Number of household members	Number of household members	Continuous variable, range: 1 to 13
Number of grandchildren under the age of 16	Number of grandchildren under the age of 16	Continuous variable, range: 0 to 14

### Empirical strategy

We move beyond descriptive, weighted OLS and exploit statutory retirement-age thresholds to identify the impact of retirement on depressive symptoms. Our core identification strategy pools men and women and instruments retirement with a single binary indicator equal to 1 if the respondent has reached the sex-specific statutory retirement age in 2018 (men ≥ 60; women ≥ 55), and 0 otherwise. We estimate IV–2SLS models in (1) a wide window covering ages 40–75 and (2) a narrower bandwidth of 55–65 centered on the statutory cutoffs, which strengthens instrument relevance. Our IV strategy follows prior studies using statutory retirement-age thresholds in China [30, 31].

All specifications adjust for parent–child satisfaction, any grandchild care, weekly care hours, self-rated health, marital status, household size, education, urban/rural residence, and a flexible age function, and include province × urban/rural fixed effects. Main-text estimates are unweighted with PSU-clustered robust SEs; survey-weighted replications are available on request. For context, we also report OLS estimates with the same covariates and fixed effects as descriptive benchmarks; these are not used for causal interpretation.

As supplementary evidence, we further estimate a male-only RDD/IV that treats turning age 60 as the instrument and allows piecewise-linear age trends on either side of the cutoff. Owing to limited power and compliance heterogeneity, the first stage is weak in this single-sex design; we therefore treat the male RDD/IV as complementary to, rather than a substitute for, the pooled sex-specific-threshold specification.

All specifications adjust for parent–child satisfaction, any grandchild care, weekly care hours, self-rated health, marital status, household size, education, urban/rural residence, and a flexible age function, and include province × urban/rural fixed effects. Main-text estimates are unweighted with PSU-clustered robust SEs; survey-weighted replications are available on request. For context, we also report OLS estimates with the same covariates and fixed effects as descriptive benchmarks; these are not used for causal interpretation.

Importantly, estimates for grandchild caregiving intensity are interpreted as conditional associations rather than causal effects, given the potential for reverse causality between depressive symptoms and caregiving capacity.

#### Baseline correlation model (Weighted OLS)

The outcome is the standardised depression score yᵢ = CES-D10. The baseline equation is

1 $${y}_{\text{i}} {\beta }^{0} + {\beta }^{1}{Retire}_{\text{i}} + {X}_{\text{i}}{\prime}\beta +f \left({Age}_{\text{i}}\right)+ {\varepsilon }_{\text{i}},$$

where Retireᵢ indicates retirement status; Xᵢ is a vector of exogenous controls. The parsimonious set includes satisfaction with children, grandchild-care provision, hours of care (HTSPCH), and self-rated health. The extended set adds pension receipt, log transfers from children, log transfers to parents, marital status, household size, number of grandchildren under 16, education, and urban/rural residence. f(Ageᵢ) is a smooth age function (see below). Because retirement may be endogenous, β₁ is interpreted as a conditional correlation only.

#### Instrument and first stage

The core instrument is

$$Zi=1\{{Age}_i\geq60\}(men\;only)$$

The first-stage equation is

2 $${Retire}_{\text{i}} = \pi 0+ {\pi 1Z}_{i}+ {X}_{i}{\prime} \pi +f \left({Age}_{i}\right)+ {u}_{i}$$

with the age trend specified as piecewise linear:

3 $$f \left({Age}_{i}\right) = \theta 1 \left({Age}_{i} -60\right)\bullet 1\left\{{Age}_{i} <60\right\}+\theta 2 \left({Age}_{i} -60\right) \bullet 1\left\{{Age}_{i} \ge 60\right\}$$

In Stata this is implemented with age_left_m/age_right_m or mkspline, absorbing smooth age effects near the cutoff and preventing any continuous trend from being mistaken for the discrete jump. Urban/rural status (urban_rural) is included as a covariate, never as an instrument. First-stage strength is gauged by the Kleibergen–Paap rk Wald F statistic and compared with Stock–Yogo critical values.

#### Second stage and parameter interpretation

Second-stage 2SLS:

4 $${y}_{\text{i}} = {\alpha }_{0} + {\alpha }_{1} {^{\wedge}\mathrm{Retire}}_{\text{i}} + {\text{X}}_{\text{i}}{\prime}\alpha +\text{f}\left({\text{Age}}_{\text{i}}\right)+ {\text{e}}_{\text{i}}$$

We estimate OLS models of standardized CES-D10 scores on retirement status and covariates (parent–child satisfaction, any grandchild care, weekly care hours, self-rated health, marital status, household size, education, urban/rural residence, and a flexible age function), including province × urban/rural fixed effects. Main-text estimates are unweighted with PSU-clustered robust SEs. Survey-weighted (svy) replications appear in Appendix Table A1.

#### Identification assumptions and tests

To mitigate endogeneity in retirement, we instrument retirement with crossing the statutory retirement-age threshold (coded 1 for men ≥ 60 and women ≥ 55, 0 otherwise). We present results for the full 40–75 window and a narrow 55–65 bandwidth. Main-text estimates are unweighted with PSU-clustered robust SEs. Because the design uses a single instrument, Hansen’s J is not reported. We report the KP F and, when relevant, Anderson–Rubin/Stock–Wright tests. First-stage diagnostics are in Appendix Table A1; LIML and survey-weighted replications are not shown and are available on request.

#### Robustness and extensions

1. Control-set progression

We begin with a “parsimonious” model that includes only Sati_Child, WPC, HTSPCH, and SRH. Controls are then expanded stepwise to encompass pension status, two-way intergenerational transfers (log_fcamt, log_tcamt), marital status, household size, education, and urban/rural residence. Stability in the magnitude and statistical significance of the retirement coefficient supports the robustness of our findings to the choice of control variables.

(2) Sample windows

We narrow the age window to 55–65 and then to 57–63, retaining the piecewise-linear age terms on both sides of the threshold. These tighter windows align the design more closely with a local fuzzy regression-discontinuity (fuzzy RD) framework.

(3) Pooled sample

Using a pooled male–female sample, we deploy a gender-specific threshold instrument.

5 $$\mathrm{Zi}\;=\;1\left\{{\mathrm{Age}}_{\mathrm i}\;\geq\;60\right\}\;\cdot1\left\{{\mathrm{Male}}_{\mathrm i}\;=1\right\}\;+\;1\left\{{\mathrm{Age}}_{\mathrm i}\;\geq55\right\}\;\cdot\;1\left\{{\mathrm{Male}}_{\mathrm i}=0\right\}\;\;$$

(4) Estimation and inference

Main-text models are unweighted with PSU-clustered robust SEs; LIML and survey-weighted replications are not shown (available on request). Weekly care hours remain capped at 168; high-influence diagnostics were checked.

(5) Nonlinearity and heterogeneity of caregiving intensity

We further examined the functional form of caregiving hours. A spline regression with a knot at 40 h per week revealed no significant effect below the threshold, but depressive symptoms increased significantly once weekly caregiving exceeded 40 h, indicating a threshold effect. In addition, we explored heterogeneity in the retirement effect by education and baseline health. Results suggest that more educated older adults are less adversely affected by retirement, while those in poorer self-rated health experience more pronounced negative impacts. These findings highlight distributional differences in retirement and caregiving effects, underscoring the importance of subgroup considerations in future research.

## Results

### Descriptive statistics

The core sample comprises 7,730 respondents, including 4,107 women and 3,623 men. The average age is 61.58 years (SD = 6.65), and mean household size is 2.82 (SD = 1.55). For health indicators, the mean CES-D10 depression score is 9.03 (SD = 3.00, raw score), and self-rated health averages 2.82 (SD = 1.03).

Among binary characteristics, 14.0% are retired (1,083/7,730), 53.5% provide grandchild care (4,137/7,730), 81.1% are married (6,268/7,730), and 75.7% reside in urban areas (5,854/7,730). Caregivers report an average of 46.6 weekly hours of care (SD = 60.8), with substantial variation.

Gender comparisons show that men are slightly older (62.43 vs. 60.83 years) but report lower depressive symptoms (mean CES-D10: 7.69 vs. 10.20). Men also have higher retirement rates (16.3% vs. 12.0%) and are more likely to be married (88.5% vs. 74.5%), whereas women are slightly more urban (77.0% vs. 74.2%). Caregiving prevalence is similar across genders (women: 53.8%; men: 53.2%), though women spend more time providing care.

Stratified results reveal that non-caregivers have the highest CES-D10 scores (women: 10.96; men: 7.93), while high-intensity caregivers report better mental health (women: 9.51; men: 7.47). Self-rated health and family size increase with caregiving intensity.

All statistics are unweighted. Continuous variables are reported as mean (standard deviation), and binary variables as percentages with counts in parentheses. Full details are presented in Table 3.

**Table 3:** Descriptive statistics for the core sample

Characteristic	No care (Women)	No care (Men)	Low intensity (< 40 h, Women)	Low intensity (< 40 h, Men)	High intensity (≥ 40 h, Women)	High intensity (≥ 40 h, Men)	Total (Women)	Total (Men)	Total (All)
CES-D10, mean (SD)	10.96 (2.83)	7.93 (3.07)	9.66 (2.98)	7.51 (3.04)	9.51 (3.02)	7.47 (3.13)	10.20 (2.93)	7.69 (3.09)	9.03 (3.00)
Self-rated health (SRH), mean (SD)	2.31 (1.00)	2.41 (1.05)	2.91 (1.00)	2.88 (1.09)	3.34 (1.01)	3.35 (1.01)	2.80 (1.01)	2.85 (1.04)	2.82 (1.03)
Age, mean (SD)	62.58 (7.21)	64.00 (6.26)	60.77 (6.77)	62.06 (5.64)	58.80 (6.54)	60.69 (5.94)	60.83 (6.93)	62.43 (6.06)	61.58 (6.65)
Family size, mean (SD)	2.31 (1.21)	2.41 (1.22)	2.91 (1.61)	2.88 (1.55)	3.34 (1.70)	3.35 (1.71)	2.80 (1.56)	2.85 (1.54)	2.82 (1.55)
Retired, % (N)	9.7 (184)	15.7 (267)	15.6 (94)	18.7 (92)	13.4 (215)	16.1 (231)	12.0 (493)	16.3 (590)	14.0 (1,083)
Providing grandchild care, % (N)	0	0	100 (602)	100 (492)	100 (1,609)	100 (1,434)	53.8 (2,211)	53.2 (1,926)	53.5 (4,137)
Married, % (N)	70.6 (1,339)	83.3 (1,414)	66.4 (400)	84.3 (415)	82.2 (1,322)	96.0 (1,377)	74.5 (3,061)	88.5 (3,207)	81.1 (6,268)
Urban resident, % (N)	78.8 (1,495)	77.2 (1,310)	74.4 (448)	70.7 (348)	75.9 (1,222)	71.9 (1,031)	77.0 (3,165)	74.2 (2,689)	75.7 (5,854)
N	1,896	1,697	602	492	1,609	1,434	4,107	3,623	7,730

*CES-D10* Center for Epidemiologic Studies Depression Scale, 10-item version. Raw CES-D10 scores (range: 0–30) are reported here for descriptive statistics, with higher values indicating more depressive symptoms; regression analyses use standardized CES-D10 scores (z-scores; range approximately –1.33 to 3.24). SRH = self-rated health, assessed on a 5-point Likert scale from 1 (poor) to 5 (excellent). Continuous variables are presented as mean (standard deviation). Categorical variables are presented as percentages with the number of respondents in parentheses. Retirement, caregiving, marital, and urban residence variables are binary indicators coded as 1 = yes and 0 = no. Caregiving status is categorized as no care, low-intensity care (< 40 h per week), and high-intensity care (≥ 40 h per week). Weekly caregiving hours (mean = 46.6, SD = 60.8 among caregivers) are not shown in the table but calculated separately for descriptive purposes

In the subsample with complete transfer data (*N* = 2,657), the mean downward financial transfer to children (log_fcamt) was 7.96 (SD = 1.37), and the mean upward transfer from children (log_tcamt) was 7.33 (SD = 1.90). Stratified by caregiving status, caregivers (wpc = 1) reported higher downward transfers (M = 8.05, SD = 1.36) than non-caregivers (M = 7.84, SD = 1.37) and similarly higher upward transfers (M = 7.45, SD = 1.80 vs. M = 7.18, SD = 2.01). By retirement status, retirees (retire = 1) reported higher downward transfers to children (M = 8.18, SD = 1.29 vs. M = 7.91, SD = 1.38) and notably higher upward transfers from children (M = 8.24, SD = 1.64 vs. M = 7.13, SD = 1.89). These patterns indicate stronger reciprocal financial exchanges among caregivers and retirees, which may partially mitigate mental health stressors. However, sample size constraints limit their inclusion in the main regression models (see Appendix Table A3).

### Regression results

Adjusting for satisfaction with children, any grandchild care, weekly caregiving hours, self-rated health, marital status, household size, education, urban/rural residence, and age, retirement is associated with lower depression: retirees score on average 0.191 SD lower on the CES-D10 than non-retirees (SE = 0.034, *p* < 0.001). Consistent with prior work, better self-rated health (coef ≈ − 0.342, *p* < 0.001) and being married (coef ≈ − 0.180, *p* < 0.001) are both linked to lower depression. Notably, higher levels of parent–child satisfaction emerge as a robust protective factor, with each one-unit increase associated with an average 0.20 SD reduction in depressive symptoms (*p* < 0.001). For caregiving, the extensive margin (any grandchild care) correlates with lower depression (coef ≈ − 0.114, *p* < 0.001), whereas the intensive margin shows a small positive gradient: each additional 10 h/week is associated with about + 0.006 SD (*p* = 0.007), suggesting opposite psychological correlates at the extensive versus intensive margins. The model explains roughly 22% of the variance (R^2^ ≈ 0.22). Full OLS results are reported in Table 4.

**Table 4:** Regression results—OLS regression

Variable	Coefficient	Standard Error	t-Statistic	*p*-Value	95% Confidence Interval
Retired	−0.191	0.034	−5.63	< 0.001	[−0.258, −0.125]
Parent–child satisfaction	−0.204	0.015	−13.45	< 0.001	[−0.233, −0.174]
Provides grandchild care (any)	−0.114	0.029	−3.89	< 0.001	[−0.171, −0.056]
Weekly caregiving hours	0.000632	0.000233	2.71	0.007	[0.000175,0.001089]
Self-rated health (1–5)	−0.342	0.01	−33.3	< 0.001	[−0.362, −0.322]
Married	−0.18	0.029	−6.19	< 0.001	[−0.237, −0.123]
Household size	−0.028	0.007	−4.11	< 0.001	[−0.041, −0.015]
Education	−0.127	0.011	−11.99	< 0.001	[−0.147, −0.106]
Urban	0.082	0.028	2.94	0.003	[0.027, 0.137]
Age	−0.010953	0.001482	−7.39	< 0.001	[−0.013858, −0.008048]
Constant	2.958	0.124	23.89	< 0.001	[2.716, 3.201]

Unweighted estimates. Robust standard errors clustered at the primary sampling unit (PSU/community). All models include province × urban/rural fixed effects and the covariates listed in §2.4.3. IV models use a single instrument (crossing the sex-specific statutory age threshold); over-identification tests are not applicable. Weak-IV diagnostics (Kleibergen–Paap rk Wald F) and weak-IV-robust tests (Anderson–Rubin, Stock–Wright) are reported in the text; see Appendix Table A1 for first-stage diagnostics

^***^ *p* < 0.01

^**^ *p* < 0.05

^*^ *p* < 0.10

#### Instrumental-Variable (IV-2SLS) results

To address potential endogeneity in retirement, we use crossing the statutory retirement age as an instrument—coded 1 for men aged ≥ 60 and for women aged ≥ 55, and 0 otherwise.

In the full sample (ages 40–75), the first-stage Kleibergen–Paap rk Wald F is ≈9.16 (borderline strength), yet the Anderson–Rubin and Stock–Wright weak-IV-robust tests are highly significant (p≈0.0001), indicating a positive effect of retirement on depressive symptoms under weak-IV-robust inference. The second-stage 2SLS estimate suggests that retirement increases the standardized CES-D10 score by about 4.04 SD units (SE = 1.74, *p* = 0.020). Given the modest instrument strength in this window, we emphasize sign and statistical significance rather than the exact magnitude.Full coefficient estimates are reported in Table 5.

**Table 5:** IV–2SLS Estimates (Ages 40–75; Unweighted; *N* = 7,730)

Variable	Coefficient	Standard Error	Z-Statistic	*p*-Value	95% Confidence Interval
Retired (IV: Z)	4.04	1.743	2.32	0.02	[0.623, 7.458]
Parent–child satisfaction	−0.189	0.025	−7.58	< 0.001	[−0.238, −0.140]
Provides grandchild care (any)	−0.283	0.085	−3.31	0.001	[−0.450, −0.115]
Weekly caregiving hours	0.001132	0.000447	2.53	0.011	[0.000256, 0.002007]
Self-rated health (1–5)	−0.373	0.021	−17.95	< 0.001	[−0.414, −0.332]
Married	−0.37	0.089	−4.13	< 0.001	[−0.545, −0.194]
Household size	0.000733	0.01606	0.05	0.964	[−0.030744, 0.032210]
Education	−0.435	0.128	−3.4	0.001	[−0.687, −0.184]
Urban	1.699	0.67	2.54	0.011	[0.386, 3.012]
Age	−0.05741	0.019235	−2.98	0.003	[−0.095110, −0.019711]
Constant	4.807	0.782	6.15	< 0.001	[3.275, 6.339]

Unweighted estimates. Robust standard errors clustered at the primary sampling unit (PSU/community). All models include province × urban/rural fixed effects and the covariates listed in §2.4.3. IV models use a single instrument (crossing the sex-specific statutory age threshold); over-identification tests are not applicable. Weak-IV diagnostics (Kleibergen–Paap rk Wald F) and weak-IV-robust tests (Anderson–Rubin, Stock–Wright) are reported in the text; see Appendix Table A1 for first-stage diagnostics

^***^ *p* < 0.01

^**^ *p* < 0.05

^*^ *p* < 0.10

Restricting the window to ages 55–65 markedly strengthens the instrument (Kleibergen–Paap F ≈ 64.83). In this bandwidth, 2SLS indicates that retirement raises the depression score by about 1.89 SD units (SE = 0.46, *p* < 0.001), with Anderson–Rubin and Stock–Wright tests again significant. Unlike the negative OLS association, the IV-based local average treatment effect is positive, implying that individuals nudged into retirement at the statutory cutoff experience a short-run deterioration in mental health. This divergence is consistent with bad-controls or reverse-causality concerns in OLS: simple correlations may capture gains from post-retirement time reallocation (e.g., more family interaction or light caregiving), whereas the IV isolates compliers at the cutoff and the impact of an abrupt, institution-driven exit from work.

The covariates move in the same directions as in OLS: better self-rated health, being married, and greater parent–child satisfaction are all significantly associated with lower depression scores; the urban–rural term is larger in the IV estimates, reflecting compositional differences near the cutoff and the local nature of the identification. Full coefficient estimates are reported in Table 6.

**Table 6:** Bandwidth Robustness IV–2SLS Estimates (Ages 55–65; Unweighted; *N* = 3,397)

Variable	Coefficient	Standard Error	Z-Statistic	*p*-Value	95% Confidence Interval
Retired (IV: Z)	1.888	0.464	4.07	< 0.001	[0.978, 2.797]
Parent–child satisfaction	−0.198	0.027	−7.3	< 0.001	[−0.251, −0.145]
Provides grandchild care (any)	−0.163	0.054	−3	0.003	[−0.270, −0.056]
Weekly caregiving hours	0.000494	0.000411	1.2	0.229	[−0.000311, 0.001299]
Self-rated health (1–5)	−0.371	0.019	−20.02	< 0.001	[−0.407, −0.335]
Married	−0.231	0.056	−4.14	< 0.001	[−0.340, −0.122]
Household size	−0.008	0.013	−0.64	0.525	[−0.033, 0.017]
Education	−0.24	0.031	−7.84	< 0.001	[−0.300, −0.180]
Urban	0.992	0.212	4.69	< 0.001	[0.577, 1.406]
Age	−0.041	0.008	−4.87	< 0.001	[−0.058, −0.025]
Constant	4.141	0.44	9.42	< 0.001	[3.279, 5.003]

Unweighted estimates. Robust standard errors clustered at the primary sampling unit (PSU/community). All models include province × urban/rural fixed effects and the covariates listed in §2.4.3. IV models use a single instrument (crossing the sex-specific statutory age threshold); over-identification tests are not applicable. Weak-IV diagnostics (Kleibergen–Paap rk Wald F) and weak-IV-robust tests (Anderson–Rubin, Stock–Wright) are reported in the text; see Appendix Table A1 for first-stage diagnostics

^***^ *p* < 0.01

^**^ *p* < 0.05

^*^ *p* < 0.10

Across all IV specifications, parent–child satisfaction remains a robust predictor of better mental health, reinforcing its role as a key protective factor independent of potential endogeneity in retirement.

#### Supplementary Evidence from the Male RDD/IV

In the male subsample, we control for piecewise-linear age trends and use turning 60 as the instrument. Owing to limited power and heterogeneous policy compliance, the first stage is weak (Kleibergen–Paap rk Wald F ≈ 0.69), yielding imprecise 2SLS estimates (coef ≈ 8.44, SE ≈ 11.22; not significant). We therefore treat this specification as suggestive evidence only: under a strict RDD/IV setup, a single gender-specific cutoff provides insufficient instrument strength for precise causal inference. By contrast, the pooled-sample design that employs gender-specific thresholds and narrows the bandwidth delivers a much stronger first stage, making the two approaches complementary. Full coefficient estimates are reported in Table 7.

**Table 7:** Male RDD/IV (piecewise-linear age trend; *N* = 4,107; PSUs = 447)

Variable	Coefficient	Standard Error	t-Statistic	p-Value	95% Confidence Interval
Retired (IV: above60)	8.437	11.224	0.75	0.452	[−13.562, 30.435]
Parent–child satisfaction	−0.228	0.053	−4.33	< 0.001	[−0.331, −0.125]
Provides grandchild care (any)	−0.538	0.513	−1.05	0.294	[−1.544, 0.467]
Weekly caregiving hours	0.0019	0.0019	0.97	0.334	[−0.0019, 0.0056]
Self-rated health (1–5)	−0.433	0.084	−5.14	< 0.001	[−0.598, −0.268]
Married	−0.408	0.351	−1.16	0.245	[−1.096, 0.280]
Household size	0.08	0.146	0.55	0.585	[−0.207, 0.366]
Education	−0.889	1.024	−0.87	0.386	[−2.896, 1.119]
Urban	3.137	3.928	0.80	0.424	[−4.561, 10.836]
Age centered at 60 (agec_m)	−0.143	0.186	−0.77	0.439	[−0.507, 0.220]
Right × Age centered	0.126	0.186	0.67	0.50	[−0.240, 0.491]
Constant	0.402	2.793	0.14	0.886	[−5.072, 5.876]

Unweighted estimates. Robust standard errors clustered at the primary sampling unit (PSU/community). All models include province × urban/rural fixed effects and the covariates listed in §2.4.3. IV models use a single instrument (crossing the sex-specific statutory age threshold); over-identification tests are not applicable. Weak-IV diagnostics (Kleibergen–Paap rk Wald F) and weak-IV-robust tests (Anderson–Rubin, Stock–Wright) are reported in the text; see Appendix Table A1 for first-stage diagnostics

^***^ *p* < 0.01

^**^ *p* < 0.05

^*^ *p* < 0.10

We further tested the interaction between retirement and the intensity of grandchild caregiving. The coefficients of the interaction terms were not statistically significant in either OLS or IV models (see Appendix Table A2), suggesting that retirement does not significantly moderate the effect of caregiving intensity on mental health.

#### Nonlinearity of caregiving intensity and heterogeneity of retirement effects

As an extension, we examined the nonlinearity of caregiving intensity and the heterogeneity of retirement effects across subgroups. A spline regression with a knot at 40 h per week showed that caregiving had no significant impact below the threshold, but depressive symptoms increased significantly once weekly caregiving exceeded 40 h, suggesting a clear threshold effect(see Appendix Table A4). This aligns with prior evidence that excessive caregiving may undermine older adults’ psychological well-being.

We also tested whether the retirement effect varied by education and baseline health. The interaction models revealed that older adults with higher educational attainment were less adversely affected by retirement, indicating a potential buffering role of education. In contrast, those in poorer self-rated health experienced significantly stronger increases in depressive symptoms after retirement(see Appendix Table A5). These findings underscore the distributional heterogeneity of retirement and caregiving effects and highlight the need for policies that provide additional support to vulnerable subgroups.

## Discussion

This study highlights the paradoxical implications of retirement for mental health. While naïve associations indicate that retirement is linked to lower depressive symptoms, causal estimates suggest that retirement at statutory ages is associated with higher depressive symptoms among compliers (Appendix Tables A5, A6). The large IV effect sizes (e.g., 1.89 SD in the 55–65 window) likely reflect weak instrument strength, model specification, or the local nature of compliers near statutory cutoffs, and should be interpreted as directional rather than precise magnitudes. The apparent benefits of retirement in OLS models are likely driven by reverse causality or unobserved confounding, whereas abrupt, rule-based exits generate psychological costs through identity loss, uncertainty, and disruption of social roles.

At the family level, grandchild caregiving exerts a dual influence. Light involvement appears to enhance intergenerational bonds and provide a sense of purpose, thereby being associated with lower depression. However, once caregiving intensity becomes excessive, the emotional and physical strain outweighs potential benefits. A spline model confirmed this threshold effect, showing that depressive symptoms increase significantly above 40 weekly hours of care (Appendix Table A4). These patterns may reflect bidirectional influences between mental health and caregiving intensity, limiting causal inference due to potential endogeneity. Importantly, the interaction between retirement and caregiving intensity is not statistically significant (Appendix Tables A4–A5), suggesting that retirement itself does not fundamentally change the psychological risks associated with high-intensity care.

Beyond caregiving, the quality of parent–child relationships consistently provides a psychological buffer. Older adults who report more harmonious ties with their children exhibit lower depressive symptoms, even after accounting for health and socioeconomic status. This finding aligns with stress–buffer theory, highlighting that emotional closeness within families can mitigate the strains of retirement or caregiving. Additionally, descriptive contrasts of intergenerational financial transfers suggest that caregivers and retirees engage in more reciprocal exchanges, potentially buffering mental health strains (Appendix Table A3). Yet, such relational and financial support is not a substitute for structural support; in contexts where childcare services are limited, even strong family bonds may be insufficient to counterbalance the mental health burden of intensive care.

The heterogeneity of retirement effects further underscores the need for targeted support. Interaction models show that higher education and better self-rated health mitigate the adverse association of retirement with depression (Appendix Table A5), indicating that vulnerable subgroups, such as those with lower education or poorer health, face greater psychological risks. Our findings suggest that reforms raising the statutory retirement age should be accompanied by parallel investments in childcare services and mental health support. Without such safeguards, delaying retirement may adversely affect the psychological well-being of older adults. Gradual exit pathways, systematic screening and counseling, and the expansion of universal childcare and respite services are critical to offset the dual pressures of abrupt retirement and heavy caregiving, while also fostering intergenerational harmony as an additional protective layer. Given the cross-sectional design, these findings should be interpreted cautiously as associations localized to statutory retirement cutoffs.

### Policy recommendations

#### Specific and actionable recommendations

This study points to three policy priorities that directly respond to the observed findings: mitigating the cliff-edge exit at statutory retirement ages, reducing the psychological risks of intensive grandchild caregiving, and bridging the institutional divide between childcare and elder care.

First, retirement transition should be smoother and more flexible. Instead of abrupt exits from formal employment, phased or partial retirement schemes—accompanied by short-term counseling and support—can help older adults adapt more gradually to shifting roles within family and community life.

Second, high-intensity caregiving demands stronger institutional relief. Expanding affordable and licensed childcare services, along with community-based respite hubs, would allow families to redistribute care more equitably and reduce the excessive reliance on grandparents who provide over 40 h of care per week. These hubs could also integrate brief mental health screening at critical life-course thresholds, ensuring timely detection and referral.

Third, better integration of childcare and eldercare systems is essential. A unified community-level platform that coordinates service eligibility, referrals, and subsidies could reduce fragmentation, ensuring that both childcare and elder care resources are jointly planned and monitored. This would prevent older adults from being structurally pushed into intensive caregiving roles simply due to service gaps.

Taken together, these recommendations emphasize transition support, service expansion, and institutional integration. While pilot programs may vary by locality, the underlying principle is to alleviate caregiving pressure, smooth retirement shocks, and build a coordinated system that addresses the dual demands of “one old and one small.”

#### International experiences as reference

Cross-national evidence suggests that universal childcare, respite services, and flexible retirement schemes are effective in alleviating intergenerational caregiving burdens. For instance, Japan’s long-term care insurance emphasizes community-based respite and coordination; Nordic countries reduce family overreliance through universal childcare and portable subsidies; and Germany combines cash transfers with service steering tied to quality thresholds. These approaches highlight the close linkage between institutional provision and mental health protection.

For China, future policy pilots should not only draw on such experiences in integrating elder care and childcare, but also embed rigorous evaluation mechanisms. Quasi-experimental designs or rapid-cycle assessments can be used to track improvements in mental health, caregiving intensity, and service uptake, ensuring both sustainability and accountability in policy implementation.

## Limitations and future directions

This study has several limitations. First, it relies on the 2018 CHARLS cross-sectional data, which constrains causal inference. We use gender-specific statutory retirement ages as instruments, but IV estimates depend on the exclusion restriction and are best interpreted as local average treatment effects (LATE) around the cutoff, rather than universal causal effects. Therefore, our findings should be interpreted cautiously as associations rather than definitive causal effects.

Second, sample selection and missing data may introduce bias. Listwise deletion excluded 95 respondents who were older (mean age 63.6 vs. 61.6) and in poorer health (mean SRH 2.3 vs. 2.8), potentially underestimating depression among vulnerable groups (Appendix Table A6). Depression and caregiving hours are self-reported and subject to recall error, while retirement is measured as a binary event, overlooking phased or partial retirement transitions.

Third, the potential endogeneity of caregiving intensity remains unaddressed: depressive symptoms may reduce caregiving capacity, creating reverse causality. As such, caregiving coefficients should be interpreted as conditional associations rather than strict causal effects.

Fourth, variables on intergenerational financial transfers sharply reduce the sample size (*N* = 2,657) and were excluded from the main regressions, limiting exploration of their buffering role. Future studies should revisit this issue using larger samples or pooled CHARLS waves.

Fifth, we do not fully exploit the complex survey design. Although weighted and unweighted results are broadly consistent, coefficients may shift under a full survey framework. Moreover, the IV–2SLS strategy identifies compliers near statutory thresholds, limiting external validity, and single-threshold male-only models suffer from weak instruments.

Finally, the modest effects observed in the spline specification for caregiving intensity and in interaction models for education and health (Appendix Tables A4 and A5) may reflect limited statistical power. Future research should leverage longitudinal or event-history data to capture dynamic mental-health trajectories before and after retirement, enrich caregiving measures with service access and family division of labor, and further investigate subgroup heterogeneity by education, baseline health, and other sociodemographic characteristics.

## Supplementary Information

Supplementary Material 1

## Data Availability

The CHARLS dataset analysed during the current study is publicly available from the official CHARLS repository (http://charls.pku.edu.cn/en).
