# Supplementary material for: Retirement and grandchild care in China: mental health consequences and policy implications
Source: BMC Public Health. 2025 Oct 8;25:3389. doi: 10.1186/s12889-025-24774-x (PMC12505542; doi:10.1186/s12889-025-24774-x)
Supplement: Supplementary file 1 — Supplementary Material 1 [file 12889_2025_24774_MOESM1_ESM.docx]

Supplementary Material

# Supplementary Data

# The CHARLS dataset analyzed in the current study is publicly available from the official CHARLS repository at [http://charls.pku.edu.cn/en](http://charls.pku.edu.cn/en).

# Supplementary Figures and Tables

Table1: Stepwise sample construction for the core analysis cohort

| Step | Constraint (added cumulatively) | Sample size (N) |
| --- | --- | --- |
| Initial sample | Respondents aged 40–75 | 7,825 |
| Drop missing: Retirement Status | Non-missing (Retirement Status) | 7,773 |
| Drop missing: Standardized CES-D10 Depression Score | Non-missing (CES-D10 score) | 7,733 |
| Drop missing: Self-rated Health Status | Non-missing (Self-rated Health Status) | 7,730 |
| Drop missing: Parent–Child Relationship Satisfaction, Providing Grandchild Care, Caregiving Intensity, Marital Status, Household Size, Years of Schooling, Urban/Rural Residence, Age, Age Squared | All above non-missing (forms core analytic sample) | 7,730 |
| Drop missing: Pension Receipt | Non-missing (Pension) | 6,947 |
| Drop missing: Financial Support from Children (log) | Non-missing (log financial transfers from children) | 5,562 |
| Drop missing: Financial Support to Children (log) | Non-missing (log financial transfers to children) | 2,657 |

Table 2: Variable Definitions

| Variable | Definition | Measurement |
| --- | --- | --- |
| Standardized CES-D10 score | Standardized CES-D10 score, a measure of depressive symptoms in older adults. Assesses symptoms such as sadness, anxiety, and loss of interest. | Continuous variable, range: -1.325 to 3.24. Higher scores indicate more severe depressive symptoms. |
| Retirement Status | Indicator variable for whether the individual is retired. | Binary variable (1 = retired, 0 = not retired). |
| Parent-Child Relationship Satisfaction | Satisfaction with the parent-child relationship. | Ordinal variable (1 = very dissatisfied, 5 = very satisfied). |
| Grandchild Caregiving | Indicates whether the individual provides caregiving to grandchildren. | Binary variable (1 = provides care, 0 = does not provide care). |
| Caregiving Intensity | Number of hours per week spent providing caregiving to grandchildren, reflecting caregiving intensity. | Continuous variable, measured in hours per week, range 0–336; values above 168 top-coded at 168. |
| Self-rated health status | Self-rated health status, reflecting the individual’s subjective health perception. | Ordinal variable (1 = very poor, 5 = excellent). |
| Pension Status | Indicates whether the individual receives a pension. | Binary variable (1 = receives pension, 0 = does not receive pension). |
| Financial Support to Children | Log-transformed amount of financial support provided to children. | Continuous variable, log-transformed financial support amount. |
| Financial Support from Children | Log-transformed amount of financial support received from children. | Continuous variable, log-transformed financial support amount. |
| The individual’s age. | The individual’s age. | Continuous variable, measured in years. |
| Squared term of age to account for non-linear age effects. | Squared term of age to account for non-linear age effects. | Continuous variable, the square of c.age. |
| Marital status. | Marital status. | Binary variable (1 = married, 0 = not married). |
| Number of household members | Number of household members. | Continuous variable, range: 1 to 13. |
| Number of grandchildren under the age of 16 | Number of grandchildren under the age of 16. | Continuous variable, range: 0 to 14. |

Note: *** p < 0.01, ** p < 0.05, * p < 0.10.

Table 3: Descriptive statistics for the core sample

| Characteristic | No care (Women) | No care (Men) | Low intensity (<40h, Women) | Low intensity (<40h, Men) | High intensity (≥40h, Women) | High intensity (≥40h, Men) | Total (Women) | Total (Men) | Total (All) |
| --- | --- | --- | --- | --- | --- | --- | --- | --- | --- |
| CES-D10, mean (SD) | 10.96 (2.83) | 7.93 (3.07) | 9.66 (2.98) | 7.51 (3.04) | 9.51 (3.02) | 7.47 (3.13) | 10.20 (2.93) | 7.69 (3.09) | 9.03 (3.00) |
| Self-rated health (SRH), mean (SD) | 2.31 (1.00) | 2.41 (1.05) | 2.91 (1.00) | 2.88 (1.09) | 3.34 (1.01) | 3.35 (1.01) | 2.80 (1.01) | 2.85 (1.04) | 2.82 (1.03) |
| Age, mean (SD) | 62.58 (7.21) | 64.00 (6.26) | 60.77 (6.77) | 62.06 (5.64) | 58.80 (6.54) | 60.69 (5.94) | 60.83 (6.93) | 62.43 (6.06) | 61.58 (6.65) |
| Family size, mean (SD) | 2.31 (1.21) | 2.41 (1.22) | 2.91 (1.61) | 2.88 (1.55) | 3.34 (1.70) | 3.35 (1.71) | 2.80 (1.56) | 2.85 (1.54) | 2.82 (1.55) |
| Retired, % (N) | 9.7 (184) | 15.7 (267) | 15.6 (94) | 18.7 (92) | 13.4 (215) | 16.1 (231) | 12.0 (493) | 16.3 (590) | 14.0 (1,083) |
| Providing grandchild care, % (N) | 0 | 0 | 100 (602) | 100 (492) | 100 (1,609) | 100 (1,434) | 53.8 (2,211) | 53.2 (1,926) | 53.5 (4,137) |
| Married, % (N) | 70.6 (1,339) | 83.3 (1,414) | 66.4 (400) | 84.3 (415) | 82.2 (1,322) | 96.0 (1,377) | 74.5 (3,061) | 88.5 (3,207) | 81.1 (6,268) |
| Urban resident, % (N) | 78.8 (1,495) | 77.2 (1,310) | 74.4 (448) | 70.7 (348) | 75.9 (1,222) | 71.9 (1,031) | 77.0 (3,165) | 74.2 (2,689) | 75.7 (5,854) |
| N | 1,896 | 1,697 | 602 | 492 | 1,609 | 1,434 | 4,107 | 3,623 | 7,730 |

Notes:CES-D10 = Center for Epidemiologic Studies Depression Scale, 10-item version. Raw CES-D10 scores (range: 0–30) are reported here for descriptive statistics, with higher values indicating more depressive symptoms; regression analyses use standardized CES-D10 scores (z-scores; range approximately –1.33 to 3.24). SRH = self-rated health, assessed on a 5-point Likert scale from 1 (poor) to 5 (excellent). Continuous variables are presented as mean (standard deviation). Categorical variables are presented as percentages with the number of respondents in parentheses. Retirement, caregiving, marital, and urban residence variables are binary indicators coded as 1 = yes and 0 = no. Caregiving status is categorized as no care, low-intensity care (<40 hours per week), and high-intensity care (≥40 hours per week). Weekly caregiving hours (mean = 46.6, SD = 60.8 among caregivers) are not shown in the table but calculated separately for descriptive purposes.

Table4: Regression Results - OLS Regression

| Variable | Coefficient | Standard Error | t-Statistic | p-Value | 95% Confidence Interval |
| --- | --- | --- | --- | --- | --- |
| Retired | -0.191 | 0.034 | -5.63 | <0.001 | [-0.258, -0.125] |
| Parent–child satisfaction | -0.204 | 0.015 | -13.45 | <0.001 | [-0.233, -0.174] |
| Provides grandchild care (any) | -0.114 | 0.029 | -3.89 | <0.001 | [-0.171, -0.056] |
| Weekly caregiving hours | 0.000632 | 0.000233 | 2.71 | 0.007 | [0.000175,0.001089] |
| Self-rated health (1–5) | -0.342 | 0.01 | -33.3 | <0.001 | [-0.362, -0.322] |
| Married | -0.18 | 0.029 | -6.19 | <0.001 | [-0.237, -0.123] |
| Household size | -0.028 | 0.007 | -4.11 | <0.001 | [-0.041, -0.015] |
| Education | -0.127 | 0.011 | -11.99 | <0.001 | [-0.147, -0.106] |
| Urban | 0.082 | 0.028 | 2.94 | 0.003 | [0.027, 0.137] |
| Age | -0.010953 | 0.001482 | -7.39 | <0.001 | [-0.013858, -0.008048] |
| Constant | 2.958 | 0.124 | 23.89 | <0.001 | [2.716, 3.201] |

Note: Unweighted estimates. Robust standard errors clustered at the primary sampling unit (PSU/community). All models include province × urban/rural fixed effects and the covariates listed in §2.4.3. IV models use a single instrument (crossing the sex-specific statutory age threshold); over-identification tests are not applicable. Weak-IV diagnostics (Kleibergen–Paap rk Wald F) and weak-IV-robust tests (Anderson–Rubin, Stock–Wright) are reported in the text; see Appendix Table A1 for first-stage diagnostics. *** p < 0.01, ** p < 0.05, * p < 0.10.

Table 5: IV–2SLS Estimates ( Ages 40–75; Unweighted; N = 7,730)

| Variable | Coefficient | Standard Error | Z-Statistic | p-Value | 95% Confidence Interval |
| --- | --- | --- | --- | --- | --- |
| Retired (IV: Z) | 4.04 | 1.743 | 2.32 | 0.02 | [0.623, 7.458] |
| Parent–child satisfaction | -0.189 | 0.025 | -7.58 | <0.001 | [-0.238, -0.140] |
| Provides grandchild care (any) | -0.283 | 0.085 | -3.31 | 0.001 | [-0.450, -0.115] |
| Weekly caregiving hours | 0.001132 | 0.000447 | 2.53 | 0.011 | [0.000256, 0.002007] |
| Self-rated health (1–5) | -0.373 | 0.021 | -17.95 | <0.001 | [-0.414, -0.332] |
| Married | -0.37 | 0.089 | -4.13 | <0.001 | [-0.545, -0.194] |
| Household size | 0.000733 | 0.01606 | 0.05 | 0.964 | [-0.030744, 0.032210] |
| Education | -0.435 | 0.128 | -3.4 | 0.001 | [-0.687, -0.184] |
| Urban | 1.699 | 0.67 | 2.54 | 0.011 | [0.386, 3.012] |
| Age | -0.05741 | 0.019235 | -2.98 | 0.003 | [-0.095110, -0.019711] |
| Constant | 4.807 | 0.782 | 6.15 | <0.001 | [3.275, 6.339] |

Note: Unweighted estimates. Robust standard errors clustered at the primary sampling unit (PSU/community). All models include province × urban/rural fixed effects and the covariates listed in §2.4.3. IV models use a single instrument (crossing the sex-specific statutory age threshold); over-identification tests are not applicable. Weak-IV diagnostics (Kleibergen–Paap rk Wald F) and weak-IV-robust tests (Anderson–Rubin, Stock–Wright) are reported in the text; see Appendix Table A1 for first-stage diagnostics.*** p < 0.01, ** p < 0.05, * p < 0.10.

Table 6:Bandwidth Robustness IV–2SLS Estimates (Ages 55–65; Unweighted; N = 3,397)

| Variable | Coefficient | Standard Error | Z-Statistic | p-Value | 95% Confidence Interval |
| --- | --- | --- | --- | --- | --- |
| Retired (IV: Z) | 1.888 | 0.464 | 4.07 | <0.001 | [0.978, 2.797] |
| Parent–child satisfaction | -0.198 | 0.027 | -7.3 | <0.001 | [-0.251, -0.145] |
| Provides grandchild care (any) | -0.163 | 0.054 | -3 | 0.003 | [-0.270, -0.056] |
| Weekly caregiving hours | 0.000494 | 0.000411 | 1.2 | 0.229 | [-0.000311, 0.001299] |
| Self-rated health (1–5) | -0.371 | 0.019 | -20.02 | <0.001 | [-0.407, -0.335] |
| Married | -0.231 | 0.056 | -4.14 | <0.001 | [-0.340, -0.122] |
| Household size | -0.008 | 0.013 | -0.64 | 0.525 | [-0.033, 0.017] |
| Education | -0.24 | 0.031 | -7.84 | <0.001 | [-0.300, -0.180] |
| Urban | 0.992 | 0.212 | 4.69 | <0.001 | [0.577, 1.406] |
| Age | -0.041 | 0.008 | -4.87 | <0.001 | [-0.058, -0.025] |
| Constant | 4.141 | 0.44 | 9.42 | <0.001 | [3.279, 5.003] |

Note:Unweighted estimates. Robust standard errors clustered at the primary sampling unit (PSU/community). All models include province × urban/rural fixed effects and the covariates listed in §2.4.3. IV models use a single instrument (crossing the sex-specific statutory age threshold); over-identification tests are not applicable. Weak-IV diagnostics (Kleibergen–Paap rk Wald F) and weak-IV-robust tests (Anderson–Rubin, Stock–Wright) are reported in the text; see Appendix Table A1 for first-stage diagnostics.*** p < 0.01, ** p < 0.05, * p < 0.10.

Table 7 Male RDD/IV ( piecewise-linear age trend; N = 4,107; PSUs = 447)

| Variable | Coefficient | Standard Error | t-Statistic | p-Value | 95% Confidence Interval |
| --- | --- | --- | --- | --- | --- |
| Retired (IV: above60) | 8.437 | 11.224 | 0.75 | 0.452 | [-13.562, 30.435] |
| Parent–child satisfaction | -0.228 | 0.053 | -4.33 | <0.001 | [-0.331, -0.125] |
| Provides grandchild care (any) | -0.538 | 0.513 | -1.05 | 0.294 | [-1.544, 0.467] |
| Weekly caregiving hours | 0.0019 | 0.0019 | 0.97 | 0.334 | [-0.0019, 0.0056] |
| Self-rated health (1–5) | -0.433 | 0.084 | -5.14 | <0.001 | [-0.598, -0.268] |
| Married | -0.408 | 0.351 | -1.16 | 0.245 | [-1.096, 0.280] |
| Household size | 0.08 | 0.146 | 0.55 | 0.585 | [-0.207, 0.366] |
| Education | -0.889 | 1.024 | -0.87 | 0.386 | [-2.896, 1.119] |
| Urban | 3.137 | 3.928 | 0.80 | 0.424 | [-4.561, 10.836] |
| Age centered at 60 (agec_m) | -0.143 | 0.186 | -0.77 | 0.439 | [-0.507, 0.220] |
| Right × Age centered | 0.126 | 0.186 | 0.67 | 0.50 | [-0.240, 0.491] |
| Constant | 0.402 | 2.793 | 0.14 | 0.886 | [-5.072, 5.876] |

Note:Unweighted estimates. Robust standard errors clustered at the primary sampling unit (PSU/community). All models include province × urban/rural fixed effects and the covariates listed in §2.4.3. IV models use a single instrument (crossing the sex-specific statutory age threshold); over-identification tests are not applicable. Weak-IV diagnostics (Kleibergen–Paap rk Wald F) and weak-IV-robust tests (Anderson–Rubin, Stock–Wright) are reported in the text; see Appendix Table A1 for first-stage diagnostics.*** p < 0.01, ** p < 0.05, * p < 0.10.

Appendix Table A1. First-stage diagnostics for IV specifications

| Window | N | KP rk Wald F | Anderson–Rubin test (p) | Stock–Wright S statistic (p) | Note |
| --- | --- | --- | --- | --- | --- |
| Ages 40–75 | 7,730 | 9.16 | < 0.001 | < 0.001 | Borderline relevance; weak-IV-robust tests significant |
| Ages 55–65 | 3,397 | 64.83 | < 0.001 | < 0.001 | Strong first stage; narrow bandwidth |

Notes: KP = Kleibergen–Paap rk Wald F statistic. AR/Stock–Wright p-values are from weak-IV-robust inference. Specifications match Tables 5–6 (unweighted; PSU-clustered SEs; covariates and fixed effects per §2.4.3).

## Appendix TableA2.Interaction between retirement and grandchild caregiving (OLS and IV estimates)

| Variables | OLS coef. (SE) | IV coef. (SE) |
| --- | --- | --- |
| Retired (yes=1) | -0.256*** (0.046) | 7.852 (7.025) |
| Hours of grandchild caregiving (per week) | -0.0003 (0.0002) | 0.006 (0.006) |
| Retired × Caregiving hours | 0.0001 (0.0005) | -0.046 (0.040) |
| Controls | Yes | Yes |
| N | 6,947 | 6,947 |

## Notes：*** p<0.01，** p<0.05，* p<0.1

## AppendixTable A3: Descriptive Statistics for Intergenerational Transfers

| Group | Variable | Mean | SD | N |
| --- | --- | --- | --- | --- |
| Non-caregivers (wpc=0) | Financial Support from Children | 7.84 | 1.37 | 1,195 |
| Non-caregivers (wpc=0) | Financial Support to Children | 7.18 | 2.01 | 1,195 |
| Caregivers (wpc=1) | Financial Support from Children | 8.05 | 1.36 | 1,462 |
| Caregivers (wpc=1) | Financial Support to Children | 7.45 | 1.8 | 1,462 |
| Non-retirees (retire=0) | Financial Support from Children | 7.91 | 1.38 | 2,196 |
| Non-retirees (retire=0) | Financial Support to Children | 7.13 | 1.89 | 2,196 |
| Retirees (retire=1) | Financial Support from Children | 8.18 | 1.29 | 461 |
| Retirees (retire=1) | Financial Support to Children | 8.24 | 1.64 | 461 |

## Note: Unweighted estimates for subsample with complete transfer data (N=2,657).

##

## AppendixTable A4. OLS with Spline for Caregiving Intensity

| Variable | Coefficient | SE | t | p-value | 95% CI |
| --- | --- | --- | --- | --- | --- |
| Retire | -0.19 | 0.03 | -5.62 | 0.000 | [-0.26, -0.12] |
| Parent–child satisfaction | -0.20 | 0.02 | -13.44 | 0.000 | [-0.23, -0.17] |
| Provides grandchild care | -0.09 | 0.05 | -2.01 | 0.044 | [-0.18, -0.00] |
| Caregiving hours (0–40) | 0.00 | 0.00 | -0.20 | 0.839 | [-0.00, 0.00] |
| Caregiving hours (>40) | 0.00 | 0.00 | 2.47 | 0.013 | [0.00, 0.00] |
| Self-rated health | -0.34 | 0.01 | -33.29 | 0.000 | [-0.36, -0.32] |
| Married | -0.18 | 0.03 | -6.19 | 0.000 | [-0.24, -0.12] |
| Household size | -0.03 | 0.01 | -4.08 | 0.000 | [-0.04, -0.01] |
| Education | -0.13 | 0.01 | -11.98 | 0.000 | [-0.15, -0.11] |
| Urban | 0.08 | 0.03 | 2.94 | 0.003 | [0.03, 0.14] |
| Age | -0.01 | 0 | -7.42 | 0.000 | [-0.01, -0.01] |
| Constant | 2.96 | 0.12 | 23.9 | 0.000 | [2.72, 3.20] |

## Note: Unweighted OLS with robust standard errors. N=7,730, R²=0.22. A spline specification with a knot at 40 weekly caregiving hours was used.

## Appendix Table A5. OLS with Interactions for Education and Health

| Variable | Model 1 (Education) | Model 2 (Health) |
| --- | --- | --- |
| Retire | -0.35 (0.08, 0.000) | -0.37 (0.10, 0.000) |
| Retire × Education | 0.06 (0.03, 0.027) |  |
| Retire × Self-rated health |  | 0.06 (0.03, 0.041) |
| Parent–child satisfaction | -0.20 (0.02, 0.000) | -0.20 (0.02, 0.000) |
| Provides grandchild care | -0.11 (0.03, 0.000) | -0.11 (0.03, 0.000) |
| Caregiving hours | 0.00 (0.00, 0.007) | 0.00 (0.00, 0.006) |
| Self-rated health | -0.34 (0.01, 0.000) | -0.35 (0.01, 0.000) |
| Married | -0.18 (0.03, 0.000) | -0.18 (0.03, 0.000) |
| Household size | -0.03 (0.01, 0.000) | -0.03 (0.01, 0.000) |
| Education | -0.14 (0.01, 0.000) | -0.13 (0.01, 0.000) |
| Urban | 0.09 (0.03, 0.002) | 0.08 (0.03, 0.004) |
| Age | -0.01 (0.00, 0.000) | -0.01 (0.00, 0.000) |
| Constant | 2.98 (0.12, 0.000) | 2.98 (0.12, 0.000) |

## Note: Unweighted OLS with robust standard errors. N=7,730, R²=0.22. Values reported as coefficient (SE, p-value). Interactions test whether retirement effects vary by education or baseline self-rated health.

## Appendix Table A6. Comparison of Included vs. Excluded Cases

| Variable | Included (N = 7,730) | Excluded (N = 95) |
| --- | --- | --- |
| Age (mean, SD) | 61.6 (7.6) | 63.6 (NA) |
| SRH (mean, SD) | 2.8 (1.0) | 2.3 (NA) |

## Note: Unweighted estimates. Standard deviation (SD) for excluded cases unavailable due to small sample size. SRH = self-rated health (1 = very poor, 5 = excellent).
